# Engineering Modular Half-Antibody Conjugated Nanoparticles for Targeting CD44v6-Expressing Cancer Cells

**DOI:** 10.3390/nano11020295

**Published:** 2021-01-23

**Authors:** Bianca N. Lourenço, Rúben F. Pereira, Cristina C. Barrias, Claudia Fischbach, Carla Oliveira, Pedro L. Granja

**Affiliations:** 1 i3S—Instituto de Investigação e Inovação em Saúde, Universidade do Porto, 4200-135 Porto, Portugal; Ruben.Pereira@ineb.up.pt (R.F.P.); ccbarrias@ineb.up.pt (C.C.B.); carlaol@ipatimup.pt (C.O.); 2 INEB—Instituto de Engenharia Biomédica, Universidade do Porto, 4200-135 Porto, Portugal; 3 IPATIMUP—Institute of Molecular Pathology and Immunology of the University of Porto, 4200-135 Porto, Portugal; 4 FEUP—Faculdade de Engenharia da Universidade do Porto, 4200-465 Porto, Portugal; 5 ICBAS—Instituto de Ciências Biomédicas Abel Salazar, Universidade do Porto, 4050-313 Porto, Portugal; 6 Nancy E. and Peter C. Meinig School of Biomedical Engineering, Cornell University, Ithaca, NY 14853, USA; cf99@cornell.edu; 7 Kavli Institute at Cornell for Nanoscale Science, Cornell University, Ithaca, NY 14853, USA; 8 Departamento de Patologia, Faculdade de Medicina da Universidade do Porto, 4200-319 Porto, Portugal

**Keywords:** gastric cancer, CD44v6, half-antibody, nanoparticles, targeting, click chemistry, bioconjugation

## Abstract

Gastric cancer (GC) remains a major cause of death worldwide mainly because of the late detection in advanced stage. Recently, we proposed CD44v6 as a relevant marker for early detection of GC, opening new avenues for GC-targeted theranostics. Here, we designed a modular nanoscale system that selectively targets CD44v6-expressing GC cells by the site-oriented conjugation of a new-engineered CD44v6 half-antibody fragment to maleimide-modified polystyrene nanoparticles (PNPs) via an efficient bioorthogonal thiol-Michael addition click chemistry. PNPs with optimal particle size (200 nm) for crossing a developed biomimetic CD44v6-associated GC stromal model were further modified with a heterobifunctional maleimide crosslinker and click conjugated to the novel CD44v6 half-antibody fragment, obtained by chemical reduction of full antibody, without affecting its bioactivity. Collectively, our results confirmed the specific targeting ability of CD44v6-PNPs to CD44v6-expressing cells (1.65-fold higher than controls), highlighting the potential of CD44v6 half-antibody conjugated nanoparticles as promising and clinically relevant tools for the early diagnosis and therapy of GC. Additionally, the rational design of our nanoscale system may be explored for the development of several other nanotechnology-based disease-targeted approaches.

## 1. Introduction

Gastric cancer (GC) is the third leading cause of mortality worldwide, within the group of malignant diseases, accounting for 8.2% of total cancer-related deaths in 2018 [1]. Despite improvements in diagnosis and therapy, the prognosis of GC patients remains poor, mostly because of late detection at advanced stage, with an overall 5-year survival rate lower than 25% [2]. Hence, there is an unmet clinical need for the development of reliable, specific, and non-invasive screening methods for early detection and therapeutic treatment of GC. Over the past decades, the field of nanomedicine has remarkably advanced, showing promise for the target-specific diagnosis and delivery of therapeutics to cancer [3,4,5]. Nonetheless, the development of GC-targeted strategies is still at its infancy [6] due, in part, to the lack of specific biomarkers for GC cells [7,8]. CD44 isoform v6 has been correlated with carcinogenesis, tumor progression, and metastasis in various cancer types [9,10,11,12,13]. In the stomach, we have shown that CD44v6 is considerably *de novo* expressed in gastric pre-malignant and malignant lesions (more than 60% of all GCs), while the normal gastric mucosa remains negative for this marker [14]. Additionally, we have recently demonstrated that CD44v6 has been correlated with poor prognosis [15] and aggressive behavior of the disease [16], suggesting its value not only for early diagnosis but also for prognosis and as a therapeutic target in GC.

A variety of ligands including antibodies, aptamers, peptides, and small biomolecules have been successfully conjugated to nanoparticles to achieve specific tumor targeting [17]. Despite the broad use of full antibodies in cancer nanomedicine [18,19,20], the common coupling chemistries used to conjugate these targeting ligands to the surface of nanoparticles, such as carboxyl-amine conjugation by carbodiimide chemistry, often result in randomly oriented grafting, leading to heterogeneity and loss of biological functionality due to steric hindrance of the antigen-binding site [21,22]. Native antibody fragments such as Fab [23,24] and half-antibody fragments [25], as well as recombinant antibody fragments including single-chain variable fragments [26], have been proposed as alternative approaches to overcome some of the pitfalls of full antibodies. Native antibody fragments are smaller in size, which results in decreased immunogenicity and allows for site-oriented conjugation, improving overall recognition efficacy [27,28]. Preferential reduction of disulfide bonds bridging the two antibody half chains, by pretreatment with reductive agents such as 2-mercaptoethylamine (2-MEA), mercaptoethanol, dithiotreitol (DTT), or tris(2-carboxyethyl)phosphine (TCEP), yields half-antibody fragments with intact antigen-binding site and reactive thiol groups in its hinge region that can be employed for site-directed conjugation [25,29,30,31,32]. In addition, this strategy can be applied to commercially available antibodies, precluding the design and production of specific antibody fragments bearing defined functional groups for site-specific bioconjugation reactions.

The aim of the present work was to develop a modular nanoscale system to selectively target CD44v6-expressing GC cells exploring, for the first time, CD44v6 half-antibody fragments as specific targeting ligands. First, to determine the most effective nanoparticle size for subsequent coupling, in vitro validation of model polystyrene nanoparticles (PNPs) transport was performed under pathologically relevant conditions using a biomimetic platform that recapitulates cellular and molecular aspects of the tumor microenvironment (e.g., 3D cell-cell and cell-ECM interactions) that serve as critical regulators of transport phenomena within tumors [33,34,35,36]. Then, a rapid and inexpensive strategy was used to obtain CD44v6 half-antibody fragments, by reducing the disulfide bonds of hinge region of CD44v6 monoclonal antibody. Bioorthogonal Michael-addition click chemistry was employed for the site-specific conjugation of CD44v6 half-antibody to maleimide-modified PNPs. Finally, the ability of the CD44v6 half-antibody-conjugated PNPs to selectively bind to CD44v6-expressing GC cells was demonstrated using an isogenic human GC cell line, previously established and explored by our group [15,16,23]. Overall, this study provides new insights for the development of more effective GC-targeting diagnostic and therapeutic approaches and emphasizes the modular and bioorthogonal nature of our strategy that can be broadly applied to develop novel targeted approaches.

## 2. Materials and Methods

### 2.1. Cell Culture

Human stomach adenocarcinoma cell line MKN74, lacking endogenous CD44 expression [37], was purchased from the Japanese Collection of Research Bioresources (JCRB) Cell Bank, while the established isogenic human GC cell line expressing the CD44v6 isoform [15] (henceforth termed as CD44v6 cells) was kindly provided by Dr. C. Oliveira, a co-author in this study. MKN74 cells (passages 49–62) and CD44v6 cells (passages 7–20) were routinely cultured in Dulbecco’s modified Eagle medium (DMEM, Gibco, Paisley, UK) containing 10% (*v*/*v*) fetal bovine serum (FBS, BioWest, Nuaillé, France) and 1% (*v*/*v*) penicillin/streptomycin (Pen/Strep, BioWest, Nuaillé, France). Additionally, the latter cells were supplemented with 1% (*v*/*v*) geneticin (Gibco, Paisley, UK). Human adipose stromal cells (ASCs, Lonza, Walkersville, MD, USA) were cultured in their corresponding growth media (ADSC-GM, Lonza, Walkersville, MD, USA) and utilized up to passage 6. Cell cultures were maintained at 37 °C under a 5% CO_2_ humidified atmosphere.

### 2.2. Preparation of Tumor-Conditioned Media

Tumor-conditioned media (TCM) was prepared as previously described [16]. In brief, MKN74 and CD44v6 cells were cultured as above until 90% confluence, rinsed with phosphate buffered saline (PBS, pH 7.4), and then incubated with low serum media (DMEM, 1% FBS, 1% Pen/Strep). After 24 h, TCM was collected, normalized to cell number, concentrated 10-fold in an Amicon centrifugal filter tube (MWCO 3 kDa, Merk Millipore, Molsheim, France), and subsequently reconstituted with low serum 1:1 DMEM/F12 containing 1% FBS and 1% Pen/Strep. Control media was incubated for 24 h in the absence of cells and then treated identically.

### 2.3. Establishment of In Vitro GC Stroma Model for Transport Studies

To define the nanoparticle size for subsequent experiments, the distribution of differently sized nanoparticles was analyzed as a function of tumor stroma-dependent transport limitations. To this end, an in vitro transport assay was developed by combining a Transwell^®^ system with a culture platform previously established by our group [16]. ASCs were seeded on the apical side of 24-well Transwell^®^ permeable inserts (PET membrane, 3 μm pore size, BD Biosciences, San Jose, CA, USA) that were previously coated with human plasma fibronectin (Sigma, St. Louis, MO, USA) at a concentration of 30 μg/mL in PBS to facilitate cell adhesion. After preconditioning cells either in control media or TCM for 7 days, Transwell^®^ membranes were washed twice in Hanks’ balanced salted solution (HBSS, Gibco, Paisley, UK) and transport studies were performed using Texas Red fluorescent carboxyl-modified polystyrene nanoparticles (PNPs, Thermo Scientific, Eugene, OR, USA) with nominal sizes of 200 and 500 nm. Afterwards, the apical side of the Transwell^®^ insert was filled with 200 μL of 100 μg/mL PNPs (either 200 or 500 nm) in HBSS, while the basolateral side was filled with 800 μL of HBSS. After 2 h of incubation at 37 °C under rotation (150 rpm), the Transwell^®^ insert was removed and the supernatant (100 μL) was transferred to a 96-well plate (black with clear bottom, Greiner). Fluorescence detection was performed using a microplate reader (Synergy MX HM550, BioTek Instruments, Winooski, VT, USA) with excitation/emission at 580/605 nm and converted into nanoparticle concentrations using a calibration curve.

### 2.4. Half-Antibody Synthesis and Characterization

Half-antibody fragments of mouse anti-human CD44v6 monoclonal antibody—clone MA54 (Thermo Scientific, Rockford, IL, USA) were obtained by reducing the disulfide bridges between the cysteine residues of the antibody in TCEP (Sigma, St. Louis, MO, USA). To optimize the reduction reaction, decreasing concentrations of TCEP solutions, serially diluted from 10 mM to 5 μM in PBS, were added to CD44v6 antibody at a final concentration of 167 μg/mL and allowed to react for 3 h at room temperature (RT). Next, to confirm the production of half-antibody fragments, the cleavage products were loaded in 10% polyacrylamide gel and separated by non-reducing SDS-PAGE (Bio-Rad, Hercules, CA, USA). The samples were run in 1X tris-glycine SDS running buffer at 150 V for 60 min (Mini Protean Tetra Cell system, Bio-Rad, Hercules, CA, USA). After electrophoresis, the resulting polyacrylamide gel was washed and stained with Coomassie Brilliant Blue R-250 (Sigma, St. Louis, MO, USA) overnight for visualization. Images were acquired using the GS-800 calibrated densitometer (Bio-Rad, Hercules, CA, USA).

### 2.5. Analysis of CD44v6 Half-Antibody Binding Affinity to CD44v6-Expressing GC Cells

Binding affinity of CD44v6 half-antibody fragments to GC cells was analyzed by flow cytometry. MKN74 and CD44v6 cells cultured in T75 flasks were harvested with Versene (Gibco, Paisley, UK) and blocked with flow cytometry buffer containing 10% FBS and 0.1% (*v*/*v*) sodium azide in PBS for 30 min on ice. Afterward, 2 × 10^5^ cells were incubated with either CD44v6 antibody (1:200) or CD44v6 half-antibody fragments (TCEP 5–1000 μM) (1:100) in flow cytometry buffer for 1 h on ice followed by three washing steps. After 1 h of incubation with anti-mouse Alexa Fluor 647-conjugated secondary antibody (Thermo Scientific, Rockford, IL, USA), cells were fixed with 4% (*v*/*v*) paraformaldehyde (PFA) for 20 min at RT and finally resuspended in PBS. Cell-associated fluorescence was measured with a FACSCalibur flow cytometer (BD Biosciences, San Jose, CA, USA) and data were analyzed with FlowJo v8.7. 

### 2.6. Synthesis of CD44v6 Half-Antibody-Conjugated Fluorescent Nanoparticles

After native antibody reduction, CD44v6 half-antibody was conjugated on the surface of carboxyl-modified PNPs with a nominal size of 200 nm through a sequential combination of aqueous carbodiimide chemistry and thiol-Michael addition click reaction. First, for the amide formation, PNPs were modified with a 3.4 kD Mal-PEG-NH_2_ heterobifunctional crosslinker (Biochempeg, Watertown, MA, USA) by carbodiimide chemistry. Briefly, 200 μg of PNPs were dispersed in 1 mL of MES buffer (0.1 M MES, 0.3 M NaCl, pH 6.5), followed by the sequential addition of 0.1 mM N-hydroxy-sulfosuccinimide (sulfo-NHS, Thermo Scientific, Rockford, IL, USA), 0.2 mM 1-ethyl-(dimethylaminopropyl)-carbodiimide (EDC, Sigma, St. Louis, MO, USA), and 0.4 mM Mal-PEG-NH_2_. After stirring for 20 h at RT in dark, the non-reacted species were removed by washing the maleimide-terminated PNPs thrice using Vivaspin 500 μL centrifugal concentrator (MWCO 300 kDa, Sartorius, Göttingen, Germany). Lastly, maleimide-PEG modified PNPs were dispersed in 15 mM HEPES buffer (pH 7.4) and covalently conjugated to the selectively reduced CD44v6 half-antibody (1000 μM TCEP) by thiol-Michael addition click reaction (molar ratio of 1:150 PNP:half-antibody). After 3 h of incubation at RT the unconjugated fragments were removed by centrifugation using the 300 kDa Vivaspin device and CD44v6-PNPs were then dispersed in PBS and stored at 4 °C protected from light until further use. 

NP size and TCEP concentration were selected based on previous results of the above-mentioned experiments. Moreover, maleimide-PEG-modified PNPs without half-antibody conjugation (CTR-PNPs) were treated similarly and used as negative control in all subsequent experiments.

### 2.7. Nanoparticle Characterization

Nanoparticle size (diameter), polydispersity index (PDI), and surface charge (zeta-potential) were measured by dynamic light scattering and laser doppler anemometry using Zetasizer (Nano ZS; Malvern, UK). CTR-PNPs and CD44v6-PNPs were dispersed in milliQ water with a final concentration of 25 μg/mL and measurements were performed at RT.

### 2.8. In Vitro Binding Studies

The targeting ability of conjugated PNPs was assessed by flow cytometry and further confirmed by immunofluorescence image analysis. Briefly, cultured cells were detached with Versene and incubated with ice-cold flow cytometry buffer for 1 h on ice. Pelleted cells (2 × 10^5^ cells/condition) were then incubated with 25 μg/mL of CTR-PNPs or CD44v6-PNPs in flow cytometry buffer for 90 min on ice, washed thrice in PBS and fixed with 4% PFA for 20 min at RT as described above. Cell-associated fluorescence of Texas Red-labeled PNPs was measured with the FACSARIA II flow cytometer (BD Biosciences, San Jose, CA, USA) and data were analyzed with FlowJo v10.

For immunocytochemistry assay, MKN74 and CD44v6 cells were plated on glass coverslips, cultured for 3 days and then incubated with 25 μg/mL of CTR-PNPs or CD44v6-PNPs in DMEM for 90 min at 37 °C. Following three washes in PBS, cells were fixed with 4% PFA for 20 min, permeabilized with 0.05% Triton X-100 in PBS for 15 min and blocked with 10% FBS in PBS for 30 min. F-actin filaments were stained with Alexa Fluor 488 phalloidin (Thermo Scientific, Rockford, IL, USA), while cell nuclei were labeled with DAPI. Imaging was performed by confocal laser scanning microscopy (CLSM SP5, Leica, Wetzlar, Germany). The scanned Z-series were projected onto a single plane and colored using Fiji software.

### 2.9. Statistical Analysis

Statistical analyses were performed using GraphPad Prism 6 software (San Diego, CA, USA). One way analysis of variance (ANOVA) followed by Bonferroni post-hoc test was applied for comparison of three or more groups, whereas two-way ANOVA with Tukey’s post-hoc test was applied for multiple comparisons. Data are represented as mean ± standard deviation (SD). For each study, three independent experiments were performed. Differences were considered statistically significant for *p* values < 0.05.

## 3. Results

### 3.1. Design of a Modular CD44v6 Half-Antibody Conjugated Nanoscale System Using a Bioorthogonal Strategy

**Figure 1:**
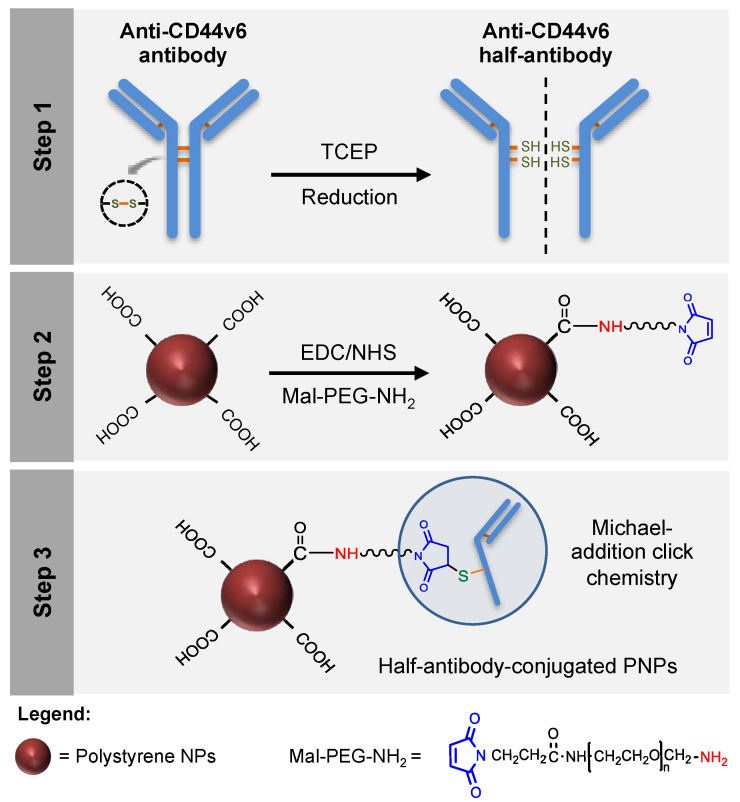
Design of a modular CD44v6 half-antibody conjugated nanoscale system using a bioorthogonal strategy. Schematic representation of the synthesis of CD44v6 half-antibody conjugated polystyrene nanoparticles (PNPs). Step 1 illustrates the selective reduction of CD44v6 antibody to half-antibody fragments using tris(2-carboxyethyl)phosphine (TCEP). Step 2 depicts the conjugation of carboxyl-terminated PNPs to maleimide-polyethylene glycol-amine (Mal-PEG-NH_2_) spacer by carbodiimide chemistry. Step 3 demonstrates the site-directed conjugation of reduced half-antibody to maleimide-PEG modified PNPs by thiol-Michael addition click chemistry, leaving the antigen-binding region free to recognize the CD44v6 receptor.

In the current study, CD44v6 half-antibody-conjugated nanoparticles were engineered through a bioorthogonal surface-engineering strategy for the specific targeting of CD44v6-expressing GC cells, as illustrated in Figure 1. The efficacy of this widely applicable strategy was demonstrated using carboxyl-modified PNPs. Although not degradable these were used as model nanoparticles because of their cytocompatibility, commercial availability, wide size range, and pliable surface properties, making them useful for conjugation of targeting ligands [38,39,40]. To create a modular system for site-specific immobilization of reduced half-antibody fragments, a combined chemical approach was followed: (1) first, carbodiimide chemistry was used to covalently bind a polyethylene glycol (PEG) linker, containing a maleimide moiety at one end and an amine group on the other (Mal-PEG-NH_2_), to PNPs surface carboxyl groups; (2) then, thiol-Michael addition click reaction was used to bind maleimides exposed at the surface of modified PNPs to free thiol groups of reduced half-antibody fragments [41]. Such bioconjugation is therefore site-specific, leaving the antigen-binding site of the half-antibody free to recognize CD44v6 receptors at GC cell surface.

### 3.2. GC-Mimetic Stroma Remodeling Hinders the Transport of PNPs in a Size-Dependent Manner

**Figure 2:**
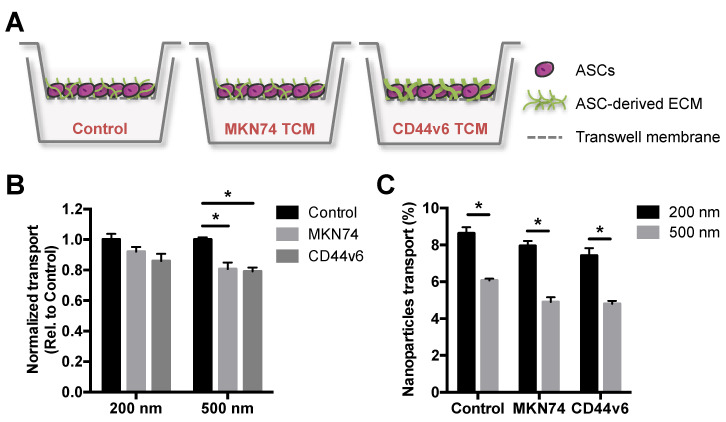
CD44v6 and MKN74 GC-mimetic stroma remodeling hinders the transport of PNPs in a size-dependent manner. (**A**) Schematic representation of in vitro gastric cancer (GC) stroma model showing adipose stromal cell (ASC)-derived extracellular matrix (ECM) treated either with control media or tumor conditioned media (TCM) from both GC cells (MKN74 TCM and CD44v6 TCM). (**B**) Quantitative analysis of 200 and 500 nm PNPs transport through in vitro GC stroma models relative to control. * *p* < 0.05. (**C**) Percentage of 200 and 500 nm PNPs that translocate the in vitro GC stroma models after 2 h of incubation. * *p* < 0.05.

Particle size plays a crucial role in the delivery of nanoparticles to solid tumors, partly due to tumor-mediated stroma remodeling that impairs transport processes by changes in ECM composition and structure [33,34,35]. Thus, we first assessed the translocation potential of PNPs with nominal sizes of 200 and 500 nm using an in vitro GC stroma model. This platform mimics cellular and ECM aspects of GC-associated stroma *in vivo* [16], and was used to select a nanoparticle size for subsequent bioconjugation that was effectively distributed in the in vitro model. The GC stroma model was generated using a Transwell^®^ system, in which human adipose stromal cells (ASCs) were cultured in the absence/presence of tumor conditioned media (TCM), from either MKN74 parental or CD44v6 cells. In the presence of CD44v6-conditioned media, ASCs undergo myofibroblast differentiation and deposit increased levels of fibrillar ECM components relative to MKN74-conditioned media [16] or control media (Figure 2A).

After 2 h of incubation with different PNPs, the effect of both ASC-mediated ECM alterations (Figure 2B) and nanoparticle size (Figure 2C) on PNPs translocation was investigated by spectroscopy. Fluorescence analysis showed that the transport of 500 nm PNPs across TCM-treated ASCs was significantly hindered, when compared to control media-treated cells, while no significant differences were observed for 200 nm (Figure 2B). Moreover, 200 nm PNPs showed enhanced translocation potential in comparison to 500 nm for all conditions (Figure 2C). Interestingly, the transport of both PNPs trended toward a decreased translocation across CD44v6-associated ECM, when compared to MKN74-associated ECM (Figure 2B), albeit these differences were not statistically significant. Collectively, these data indicate that stroma-mediated differences in ECM remodeling may contribute to a size-dependent delivery of nanoparticulate systems into tumors. Accordingly, these results suggest that smaller particle sizes should be considered for the development of nanoparticle-based GC targeting strategies.

### 3.3. Selective TCEP-Reduction of CD44v6 Antibody Generates Functional Half-Antibody Fragments

**Figure 3:**
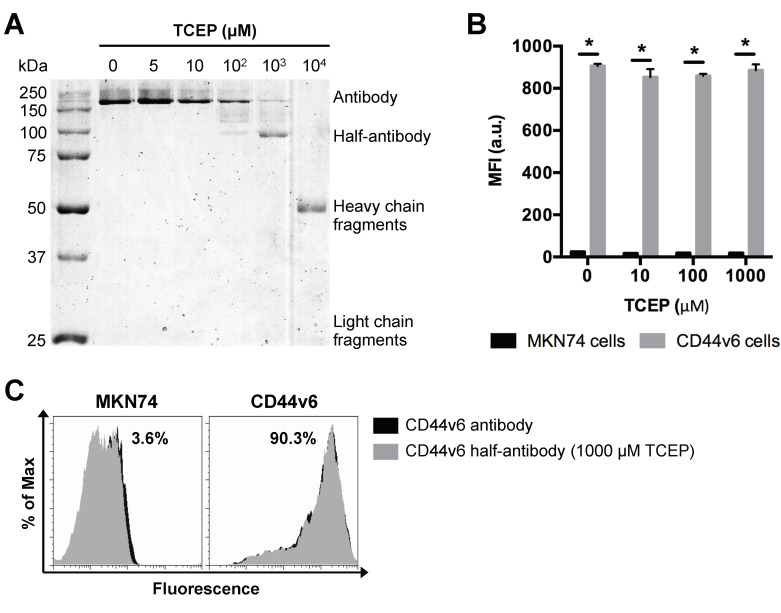
Selective TCEP-reduction of CD44v6 antibody generates half-antibody fragments that retain binding affinity to CD44v6-expressing GC cells without loss of selectivity. (**A**) SDS-PAGE analysis of mouse anti-human CD44v6 monoclonal antibody reduction products obtained using a range of TCEP concentrations. (**B**) Flow cytometry analysis of MKN74 and CD44v6 cell binding to CD44v6 antibody fragments reduced at different concentrations of TCEP. MFI = geometric mean fluorescence intensity; * *p* < 0.05. (**C**) Representative histograms of MKN74 and CD44v6 cells analyses by flow cytometry quantifying the binding affinity of CD44v6 antibody (clone M454) and 1000 μM TCEP-reduced half-antibody fragments to CD44v6 receptor.

Based on our findings above, PNPs with a nominal size of 200 nm were used as model particle to produce CD44v6 half-antibody conjugated nanoparticles. To optimize the selective reduction process and confirm the ability to generate half-antibody fragments, CD44v6 antibody was incubated with different concentrations of TCEP for 3 h and then separated by non-reducing sodium dodecyl sulfate polyacrylamide gel electrophoresis (SDS-PAGE) (Figure 3A). Image analysis of coomassie blue-stained gels confirmed the presence of intact antibody (~200 kDa due to glycosylation) for lower concentrations of TCEP (5–100 μM). In contrast, distinct cleavage products, including heavy (~50 kDa) and light chain (~25 kDa) fragments [42], were detected for the highest concentration of reducing agent (10^4^ μM TCEP). These data suggest that disulfide bridges between heavy and light chains were also reduced. Notably, when CD44v6 antibody was reduced with 1000 μM TCEP, the presence of a distinct band at ~100 kDa indicates preferential cleavage of disulfide bridges between the two heavy chains of hinge region, resulting in the formation of half-antibody fragments, as envisaged. These findings highlight the importance of optimizing TCEP concentration. As shown, 1000 μM TCEP increases the yield of CD44v6 half-antibody production, while reducing the formation of non-targeting fragments, which may compete with half-antibody fragments for maleimide conjugation sites at modified-PNPs surface.

### 3.4. TCEP-Reduced Half-Antibody Fragments Retain Binding Affinity to CD44v6-Expressing GC Cells without Loss of Selectivity

Conjugation efficiency and targeting ability of CD44v6 half-antibody conjugated PNPs depend on the extent of antibody reduction, as it determines both the density of free thiol functional groups and the integrity/functionality of antibody fragments. To verify whether the reduced half-antibody preserved its binding affinity to CD44v6-expressing cells, both MKN74 and CD44v6 cells were incubated with either untreated or TCEP-treated CD44v6 antibody and analyzed by flow cytometry. Fluorescence measurements indicated that, after the reduction step, antibodies treated with TCEP concentrations in a range of 5 μM to 1000 μM retain their antigen binding ability to CD44v6 cells, as compared to untreated antibodies (Figure 3B,C). The binding affinity of 10^4^ μM TCEP-treated antibody was not tested because of its complete cleavage. Together, these results confirmed that CD44v6 half-antibody fragments with preserved binding sites to CD44v6-expressing cells could be generated under mild conditions, using 1000 μM TCEP. Thus, this strategy makes thiol groups on the hinge region of half-antibodies available for site-orientated conjugation to maleimide-modified PNPs by click chemistry [43], while leaving antigen binding moieties free for antigen molecular recognition.

### 3.5. Clickable Surface-Engineering Strategy Allows Efficient Ligand Bioconjugation

The successful surface modification of PNPs with both Mal-PEG-NH_2_ crosslinker and half-antibody fragments was confirmed by zeta-potential measurements, before and after bioconjugation steps, as shown in Table 1. The surface charge of unmodified (bare) PNPs changed from −45.3 ± 1.1 mV to −31.4 ± 3.5 mV after maleimide modification (control, CTR-PNPs). This change was likely due to the reduction of negatively charged carboxylic groups present at the PNPs surface via amide bond formation with the heterobifunctional crosslinker. Additionally, after half-antibody conjugation, nanoparticles’ zeta-potential significantly increased to −25.1 ± 7.2 mV (CD44v6-PNPs), demonstrating the successful conjugation of half-antibody fragments at PNPs surface, mostly because of the positively charged amine groups present in the N-terminus of half-antibody chain. Similarly, dynamic light scattering (DLS) measurements showed an enhancement of mean size of PNPs after bioconjugation, from 237 ± 2 nm to 352 ± 72 nm (CD44v6-PNPs), with lower degree of nanoparticle aggregation (PDI of 0.25 ± 0.10).

### 3.6. CD44v6 Half-Antibody Conjugated PNPs Selectively Bind to CD44v6-Expressing GC Cells

**Figure 4:**
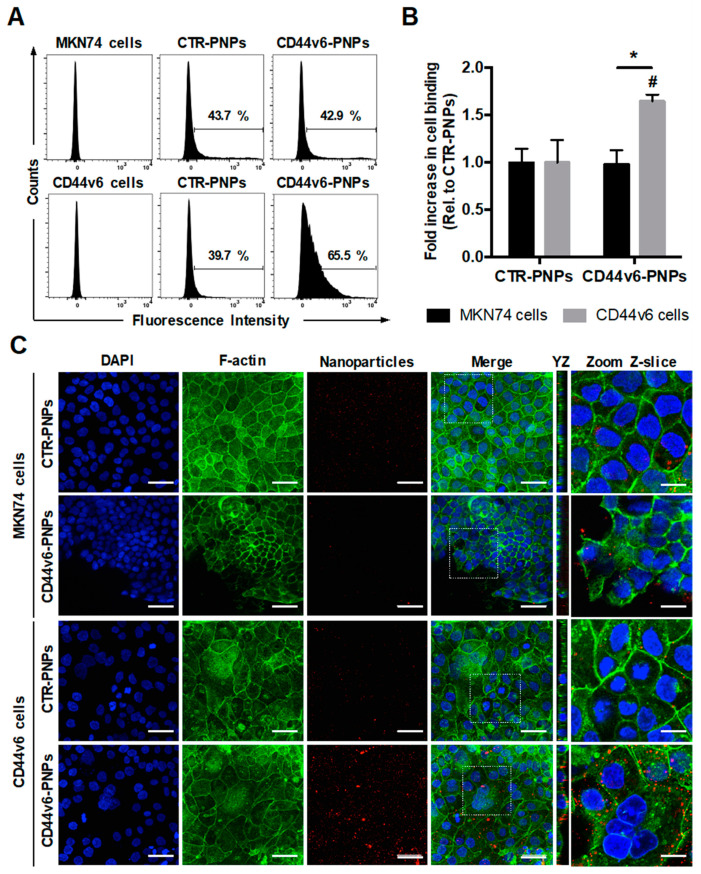
CD44v6 half-antibody conjugated PNPs selectively bind to CD44v6-expressing GC cells. Flow cytometry analysis showing (**A**) the percentage of MKN74 and CD44v6 cells binding to CTR-PNPs and CD44v6-PNPs and (**B**) the mean fold increase in cell binding over CTR-PNPs conditions after 90 min of incubation at 4 °C. A minimum of 10,000 events were evaluated for each measurement. * *p* < 0.05 from different cell lines within the same experimental condition and # *p* < 0.05 between experimental conditions for a given cell line. (**C**) Immunofluorescence images of MKN74 and CD44v6 cells incubated with 25 μg/mL of CTR-PNPs and CD44v6-PNPs at 37 °C for 90 min. Scale bars = 50 μm. The two rightmost panels correspond to YZ panels and a higher magnification of a single Z-slice confocal image acquired for each experimental condition. Scale bars = 20 μm. Nuclei (DAPI), F-actin and PNPs are stained in blue, green and red, respectively. DAPI = 4′,6-diamidino-2-phenylindole.

To verify the specificity of CD44v6-mediated targeting of conjugated PNPs to GC cells, MKN74 and CD44v6 cells were treated with 25 μg/mL of Texas Red-labeled CTR-PNPs or CD44v6-PNPs and analyzed through flow cytometry (Figure 4A). Results showed a similar shift in both GC cells treated with CTR-PNPs (43.7% for MKN74 cells and 39.7% for CD44v6 cells), suggesting unspecific NP uptake by both GC cell lines. In contrast, CD44v6-PNPs-treated CD44v6 cells displayed a statistically significant shift in the flow cytometry diagram (65.5%) in comparison to MKN74 cells (42.9%). Thus, CD44v6 half-antibody-conjugated PNPs exhibited a significant 1.65-fold increase in binding specificity to CD44v6-expressing GC cells (Figure 4B) compared with CTR-PNPs, suggesting an enhanced receptor-mediated cellular binding.

To provide additional qualitative analysis to support our findings, the cell-binding activity of Texas Red-labeled CTR-PNPs and CD44v6-PNPs was further studied by confocal fluorescence microscopy after F-actin and nuclei staining (Figure 4C). In agreement, with the flow cytometry results, after incubation of GC cells with CTR-PNPs, a very weak red signal in both cell lines was detectable, showing limited cell-NP interactions. Similarly, for MKN74 cells exposed to the CD44v6-PNPs, residual red fluorescence was detected. In contrast, when CD44v6-PNPs were added to CD44v6-expressing cells, intense red fluorescence signal was detected, suggesting that CD44v6-PNPs were not only able to specifically bind to CD44v6 cells, but also be internalized into these cells, as shown in the YZ planes of confocal images (Figure 4C).

To evaluate whether conjugated PNPs maintain their targeting ability after storage, we next tested the cell-binding activity of conjugated PNPs 3 weeks post-production by flow cytometry analysis (Figure S1). The percentage of cell binding showed to decrease with time for all the tested conditions, most likely due to PNP fluorescence lifetime, and to potential reduced structural integrity of the half-antibody as a result of the storage conditions (4 °C instead of −20 °C at which the CD44v6 monoclonal antibody is usually stored). Notably, CD44v6-PNPs still showed a trend toward increased binding to CD44v6 cells, as compared to control conditions, suggesting that both stability of thioether bond and half-antibody targeting ability are preserved during the tested period of time. Nevertheless, further investigation is warranted to define the ideal storage conditions of conjugated PNPs that ensure long-term stability required for clinical application.

Collectively, our results confirmed the successful bioconjugation of modified-PNPs maleimide groups to half-antibody thiol groups, preserving its bioactivity and leaving the antigen-binding site (Fab region) available for targeting, for up to 3 weeks post-production. Notably, our results demonstrated that half-antibody CD44v6 conjugated-PNPs selectively bind to CD44v6-expressing cells, as compared to control PNPs and cells, highlighting the receptor-mediated targeting potential of our modular half-antibody conjugated nanoscale system.

## 4. Discussion

Our previous work has demonstrated that CD44v6 is *de novo* expressed in more than 60% of GCs, being associated with poor prognosis and aggressive behavior of the disease [14,15,16]. Despite the potential of CD44v6 as a molecular target, to date just a few CD44v6-targeting ligands, including monoclonal antibodies, low molecular weight antibody fragments, and peptides have been successfully immobilized or conjugated to nanoparticles [19,23,26,44]. Therefore, novel approaches are still needed for the development of fine-tuned NP-based targeted systems exhibiting efficient and specific GC targeting. To the best of our knowledge, this is the first time that CD44v6 half-antibody conjugated nanoparticles were engineered to specifically target GC cells, though few other half-antibodies have been reported as nanoparticle-targeting ligands [25,29,30,31,32]. While covalent conjugation of full antibodies on nanoparticle surface via amine or carboxylic acid groups has been extensively studied, this approach leads to random orientation and uncontrolled coupling of antibody molecules because of their high content of reactive amine- and carboxyl-containing residues. Notably, the lack of control over antibody orientation may lead to steric hindrance of the target-binding site, limiting antibody-antigen interactions, thus resulting in decreased cell-binding efficiency [21,22]. Engineered or recombinant antibody fragments are a promising and valuable alternative to full antibodies because of controlled selection of domains, higher purity, and homogeneity. While recombinant antibodies present disadvantages in terms of high cost, variable stability, and challenging manufacturing and purification procedures, production of antibody fragments via chemical reduction of full antibodies is inexpensive, less complex, and easily performed using standard tools [45,46,47]. Importantly, half-antibody fragments, consisting of a heavy and a light chain, obtained by preferential reduction of full antibodies using mild reducing agents (e.g., 2-MEA, mercaptoethanol, DTT or TCEP) display improved stability and are easily produced when compared to Fab fragments obtained by F(ab)’2 reduction [47,48,49]. Moreover, preferential reduction of full antibodies can be applied to a wide range of immunoglobulin classes (IgG, IgA, and IgD) and species, including humanized antibodies, overcoming immunogenicity problems in the clinic.

To explore these unique attributes, this work reports a robust and widely applicable approach for the site-specific and bioorthogonal conjugation of a half-antibody to nanoparticles. The strategy combines carbodiimide and click thiol-Michael addition chemistries toward the production of CD44v6 half-antibody conjugated-nanoparticles. Herein, it was demonstrated and validated that the bioconjugation of PNPs with a TCEP-reduced CD44v6 half-antibody endows nanoparticles with the ability to target significantly more GC cells expressing CD44v6 that were previously established and reported by our group [15,16,23], than CD44v6 negative cells (MKN74 cell line). The half-antibody was engineered by selective reduction of disulfide bonds of the hinge region of CD44v6 antibody using an optimized TCEP concentration (1000 μM). Then, it was covalently linked to PNPs through a combination of carbodiimide and click Michael-addition chemistries using a Mal-PEG-NH_2_ crosslinker to form the CD44v6-PNP conjugate. Specifically, the addition of Mal-PEG-NH_2_ crosslinker into this system enabled us to fine-tune the site-directed conjugation of half-antibody fragments at PNPs surface and reduce non-specific binding of the nanoparticles to cells. Moreover, the PEG spacer also increased the structural availability of maleimides to bind thiol groups exposed on the reduced half-antibody fragments and form a stable thioether bond, precluding non-specific degradation of antibody-PEG spacer bond [50,51]. Unlike the use of thiol-based reducing agents that form to some extent disulfide adducts between the reducing agent and the thiol being activated [52], trialkylphosphine, such as TCEP, prevents the formation of thiol adducts. Additionally, since TCEP is devoid of sulfur-containing groups, it does not compete with the reduced half-antibody for maleimide binding sites on PNP surface. This implies that the excess of TCEP does not have to be removed prior functionalization with maleimide-modified PNPs [53], making it a more convenient reducing agent for this application. Albeit the successful conjugation of both Mal-PEG-NH_2_ crosslinker and half-antibody fragment was confirmed by particle size and zeta potential measurements, performed before and after bioconjugation steps, additional studies are warranted to further elucidate the influence of reaction conditions on overall conjugation efficiency.

Diffusion of nanoparticles across the abundant interstitial matrix of cancer-associated stroma constitutes the final step to target GC cells infiltrated within the gastric mucosa. Therefore, this study also focused on how varied TCM-treated stroma and thus ECM changes impact the translocation of nanoparticles of different sizes. We have recently demonstrated that CD44v6 overexpression plays a key role in the formation of a tumor-promoting microenvironment by inducing the proliferation and differentiation of ASCs into myofibroblasts, which in turn increase fibrotic/desmoplastic ECM deposition and remodeling [16]. Hence, we explored this finding to develop a GC-associated fibrotic stroma model to study the role of CD44v6 TCM-induced changes in ECM remodeling on PNP transport. While the transport of larger PNPs was significantly decreased in both GC stromal models when compared to control stromal model, the transport of 200 nm PNPs was not significantly affected by ECM alterations. Interestingly, although no statistically differences were observed, our results showed a trend toward decreased transport of both PNPs across CD44v6-associated fibrotic/desmoplastic stroma in comparison to both control conditions. This trend is also supported by other studies showing that limited transport of nanoparticles is correlated with desmoplastic response in many tumors, due to excessive ECM deposition and stiffening by myofibroblasts [33,34,35]. In addition to high ECM concentrations, desmoplastic tumors are also characterized by high stromal cell density that compresses the matrix into a dense and disorganized network, limiting convection of nanoparticles, while collagen fibrillar structure, mesh size and thickness directly limit nanoparticle diffusion [36,54]. We have recently demonstrated the active role of stomach fibroblasts, together with the complexity of the stomach *lamina propria*, mimicked by a 3D cell-laden hydrogel, as important modulators of PNP transport across the gastric mucosa [39]. Moreover, the appropriate size for efficient transport has been described to be within a range between 70 and 200 nm [55]. Smaller nanoparticles may be easily removed from tumor sites by fast blood flow, whereas nanoparticles bigger than 200 nm present low permeation efficacy into the solid tumors [55]. Accordingly, our results suggest the implementation of smaller particle sizes for optimal nanoparticle-based GC target approaches, to reduce diffusional hindrance and improve penetration into the interstitial matrix [56]. Although previous reports may support our results, future mechanistic studies will be necessary to further confirm our findings.

While nanoparticles are easily internalized by most of the cells, the overall targeting success depends on nanoparticle-uptake efficiency by appropriate pathways. Although PNPs have shown unspecific binding to different cell types [38], our results revealed that PNPs conjugated in a site-oriented manner with CD44v6 half-antibody, bind more significantly to CD44v6-expressing GC cells when compared to CD44v6 negative cells. Furthermore, similar targeting efficiency results of active targeting nanoparticles compared to non-targeted nanoparticles were reported in the literature for cancer therapy (Table S1), confirming the biological relevance of our findings. Since our isogenic model of MKN74 parental and CD44v6-expressing GC cells differ in terms of CD44v6 expression [15], with MKN74 lacking endogenously this protein [37], our data strongly suggest that CD44v6-PNPs can bind to cells in a CD44v6 receptor-mediated manner, opening new opportunities for the development of novel CD44v6-targeted drugs.

Overall, our results demonstrate the importance of developing integrated and modular strategies, based on suitable bioconjugation chemistries for the design of more effective nanoparticles for directed targeting of CD44v6-expressing cancer cells. Importantly, CD44v6 expression has been detected not only in GC, but also in a variety of other human malignancies, such as breast, lung, ovarian, and colorectal cancers [9,10,11,12,13], widening the applicability of our modular targeting nanoscale system. Future studies will focus on extending this work beyond model nanoparticles to more therapeutically relevant polymeric nanoparticles, such as poly(lactide-co-glycolide) (PLGA), polylactide (PLA), poly(D,L-lactide), polyglycolide (PGA), polycaprolactone (PCL), chitosan, and also PLGA-PEG that has shown reduced unspecific binding to GC cells [23]. Additionally, the use of Michael-addiction click reaction herein explored for site-directed maleimide-sulfhydryl PNP conjugation is compatible with a variety of proteins and biomolecules. This opens new avenues for the development of multifunctional nanoparticles [5,56,57] that may include, for instance, the design of nanoscale systems carrying therapeutic agents, but also for bio-molecular targeting and imaging signal amplification. Another relevant application could be bio-barrier avoidance by the use of permeation enhancer agents, like ECM-degrading enzymes [58,59,60]. Finally, it would also be worthy to investigate whether the developed nanoparticles can effectively adhere to and cross the mucosal barrier and target CD44v6-expressing GC cells within the gastric mucosa by exploring clinically relevant 3D tissue engineered models mimicking the tumors in vivo [39,61,62].

## 5. Conclusions

By exploring the high affinity and sulfhydryl to maleimide reactivity of newly reduced CD44v6 half-antibody fragments, we outlined an efficient bioorthogonal click strategy to site-specifically tether half-antibody fragments to maleimide-modified nanoparticles for selective targeting of CD44v6-expressing GC cells. This strategy overcomes major pitfalls of current approaches exclusively based on carboxyl-amine coupling of full antibodies, providing superior control over site-oriented ligand conjugation and leading to optimized orientation of half-antibody antigen binding site on the surface of nanoparticles in a conformation that enhances binding specificity to CD44v6-expressing GC cells. Overall, our findings open new avenues on the use of CD44v6 half-antibody fragments as an attractive and specific tumor-targeting ligand, highlighting the great potential of our strategy in the design of powerful tools for the early theranostics of GC. Additionally, the modular and multifunctional nature of the concept here reported might be exploited to a wider range of nanotechnology-based approaches, contributing to an exciting time in the development of clinically effective targeted disease therapies.

## Figures and Tables

**Table 1:** Clickable surface-engineering strategy allows efficient ligand bioconjugation. Particle size (nm), polydispersity index (PDI), and zeta potential (mV) of PNPs before and after conjugation to CD44v6 half-antibody fragments. * *p* < 0.05 relative to Bare-PNPs.

Nanoparticle	Composition	Mean Size (nm)	PDI	Zeta Potencial (mV)
Bare-PNPs	PNP	237 ± 2	0.05 ± 0.01	−45.3 ± 1.1
CTR-PNPs	PNP-PEG-MAL	309 ± 50 *	0.19 ± 0.09 *	−31.4 ± 3.5 *
CD44v6-PNPs	PNP-PEG-MAL-CD44v6	352 ± 72 *	0.25 ± 0.10 *	−25.1 ± 7.2 *

## Supplementary Materials

The following are available online at https://www.mdpi.com/2079-4991/11/2/295/s1. Table S1: Flow cytometry analysis of MKN74 and CD44v6 cell binding to CTR-PNPs and CD44v6-PNPs 3 weeks post-production stored at 4 °C in PBS. Table S1: Examples of active targeting nanoparticles for cancer therapy. Efficiency of active targeting compared to non-targeted nanoparticles reported in the literature for cancer therapy.
