# Supplementary material for: Engineering Modular Half-Antibody Conjugated Nanoparticles for Targeting CD44v6-Expressing Cancer Cells
Source: Nanomaterials (Basel). 2021 Jan 23;11(2):295. doi: 10.3390/nano11020295 (PMC7912417; doi:10.3390/nano11020295)
Supplement: Supplementary file 1 [file nanomaterials-11-00295-s001.pdf]

Supplementary Information

Engineering Modular Half-Antibody Conjugated Nanoparticles for Targeting CD44v6-Expressing Cancer Cells

Bianca N. Lourenço[1,2,3,4,*], Rúben F. Pereira[1,2,5], Cristina C. Barrias[1,2,5], Claudia Fischbach[6,7], Carla Oliveira[1,3,8], Pedro L. Granja[1,2,*]

[1]i3S - Instituto de Investigação e Inovação em Saúde, Universidade do Porto, 4200-135 Porto, Portugal

[2]INEB - Instituto de Engenharia Biomédica, Universidade do Porto, 4200-135 Porto, Portugal

[3]IPATIMUP - Institute of Molecular Pathology and Immunology of the University of Porto, 4200-135 Porto, Portugal

[4]FEUP - Faculdade de Engenharia da Universidade do Porto, 4200-465 Porto, Portugal

[5]ICBAS - Instituto de Ciências Biomédicas Abel Salazar, Universidade do Porto, 4050-313 Porto, Portugal

[6]Nancy E. and Peter C. Meinig School of Biomedical Engineering, Cornell University, Ithaca, NY 14853, USA

[7]Kavli Institute at Cornell for Nanoscale Science, Cornell University, Ithaca, NY 14853, USA

[8]Departamento de Patologia e Oncologia, Faculdade de Medicina da Universidade do Porto, 4200-319 Porto, Portugal

*Correspondence: bianca.lourenco@ineb.up.pt (B.N.L.); pgranja@i3s.up.pt (P.L.G.)

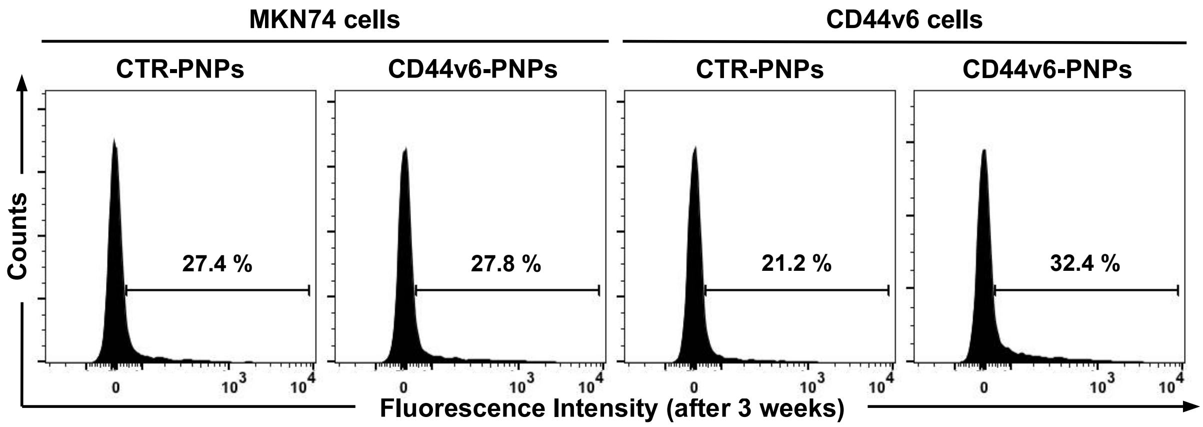

Figure S1. CD44v6 half-antibody conjugated PNPs retain binding to CD44v6 expressing GC cells after storage. Flow cytometry analysis of MKN74 and CD44v6 cell binding to CTR-PNPs and CD44v6-PNPs 3 weeks post-production stored at 4 °C in PBS.

Table S1. Examples of active targeting nanoparticles for cancer therapy. Efficiency of active targeting compared to non-targeted nanoparticles reported in the literature for cancer therapy.

Ligand	Receptor	Nanoparticle	*In vivo*/*in vitro* study	Efficiency compared to non-targeted NPs	Ref
IL-13 peptide	IL-13Rα2	Docetaxel-loaded PEG-PCL	*In vivo*: human primary glioblastoma cell line (U87) orthotopic xenograft	1.73-fold higher tumor targeting	1
CVKTPAQSC peptide	CD133	Docetaxel-loaded PLA	*In vitro:* human lung cancer cell line (A549)	1.88-fold higher cell targeting	2
cRGDyK	Integrin αvβ3	Paclitaxel-loaded micelle	*In vitro:* human prostate cancer cell line (PC-3)	1.93-fold higher cell targeting	3
RGD	Integrin αvβ3	Doxorubicin-dendritic poly-L-lysine-gelatin	*In vivo:* murine mammary carcinoma cell line (4T1) xenograft	1.18-fold higher tumor accumulation	4
Anti-CD44v6 Fab	CD44v6	Bevacizumab-loaded PLGA-PEG	*In vitro:* human stomach adenocarcinoma cell line (MKN74)	2.00-fold higher cell targeting	5
Hyaluronic acid	CD44	Paclitaxel-micelle	*In vivo:* murine hepatic carcinoma cell line (Heps) xenograft	2.80-fold higher tumor accumulation	6
Folic acid	Folate	Doxorubicin-dendrimer	*In vitro:* human epidermal carcinoma cell line (KB)	1.40-fold higher cell targeting	7
Folic acid	Folate	Gemcitabine-loaded BSA	*In vitro:* human breast adenocarcinoma cell line (MCF-7)	2.00-fold higher cell targeting	8
Folic acid	Folate	Doxorubicin-β-cyclodextrin	*In vitro:* human breast adenocarcinoma cell line (MCF-7)	1.98-fold higher cell targeting	9

Aptamer AS1411	Nucleolin	Doxorubicin-loaded HPAEG	*In vitro:* human breast adenocarcinoma cell line (MCF-7)	2.00-fold higher cell targeting	10
Aptamer AS1411	Nucleolin	Methotrexate-UnTHCPSi-PEI	*In vitro:* human breast adenocarcinoma cell line (MDA-MB-231)	1.60-fold higher cell targeting	11
Aptamer AS1411	Nucleolin	Doxorubicin-loaded polymersome	*In vitro:* human breast adenocarcinoma cell line (MCF-7)	1.73-fold higher cell targeting	12
Aptamer AS1411	Nucleolin	Doxorubicin-loaded polymersome	*In vivo:* human breast adenocarcinoma cell line (MCF-7) xenograft	1.75-fold higher cell targeting	12
Galactose	Lecithin	Doxorubicin-loaded solid lipid	*In vitro:* human lung cancer cell line (A549)	1.50-fold higher cell targeting	13
Folic acid and BSA	Folate and SPARC	Paclitaxel-loaded lipid	*In vitro:* human breast adenocarcinoma cell line (MCF-7)	1.90-fold higher cell targeting	14

Abbreviations: BSA – bovine serum albumin; cRGDyK - cyclic arginine-glycine-aspartic acid-tyrosine-lysine; Fab - antigen-binding fragment; HPAEG – hyperbranched poly(2-((2-(acryloyloxy)ethyl)disulfanyl)ethyl 4-cyano-4(((propylthio)carbonothioyl) -thio)-pentanoate-co-poly(ethylene glycol) methacrylate; IL-13 - Interleukin 13; PCL - polyethylene glycol-poly(ε-caprolactone); PEG - polyethylene glycol; PEI - polyethylenimine; PLA - poly(lactic acid); PLGA - poly(lactic-co-glycolic acid); NPs – nanoparticles; RGD - arginine–glycine–aspartic acid peptide; SPARC - secreted protein acidic and rich in cysteine; UnTHCPSi - undecylenic acid modified thermally hydrocarbonized porous silicon.

References

1. Gao H, Zhang S, Yang Z, Cao S, Jiang X, Pang Z. In vitro and in vivo intracellular distribution and anti-glioblastoma effects of docetaxel-loaded nanoparticles functioned with IL-13 peptide. *Int J Pharm.* 2014;466(1–2):8–17.
2. Yang N, JiangY, Zhang H, et al. Active targeting docetaxel-PLA nanoparticles eradicate circulating lung cancer stem-like cells and inhibit liver metastasis. *Mol Pharm.* 2015;12(1):232–239.
3. Gao Y, Zhou Y, Zhao L, et al. Enhanced antitumor efficacy by cyclic RGDyK-conjugated and paclitaxel-loaded pH-responsive polymeric micelles. *Acta Biomater*. 2015;23:127–135.
4. Hu G, Zhang H, Zhang L, Ruan S, He Q, Gao H. Integrin-mediated active tumor targeting and tumor microenvironment response dendrimer- gelatin nanoparticles for drug delivery and tumor treatment. *Int J Pharm*. 2015;496(2):1057–1068.
5. Baião A, Sousa F, Oliveira AV, Oliveira C, Sarmento B. Effective intracellular delivery of bevacizumab via PEGylated polymeric nanoparticles targeting the CD44v6 receptor in colon cancer cells. *Biomater Sci.* 2020;8:3720–3729.
6. Yin S, Huai J, Chen X, et al. Intracellular delivery and antitumor effects of a redox-responsive polymeric paclitaxel conjugate based on hyaluronic acid. *Acta Biomater*. 2015;26:274–285.

7. Cheng L, Hu Q, Cheng L, et al. Construction and evaluation of PAMAM-DOX conjugates with superior tumor recognition and intracellular acid-triggered drug release properties. *Colloids Surf B Biointerfaces*. 2015;136:37–45.
8. Dubey RD, Alam N, Saneja A, et al. Development and evaluation of folate functionalized albumin nanoparticles for targeted delivery of gemcitabine. *Int J Pharm*. 2015;492(1–2):80–91.
9. Yin JJ, Sharma S, Shumyak SP, et al. Synthesis and biological evalu- ation of novel folic acid receptor-targeted, β-cyclodextrin-based drug complexes for cancer treatment. *PLoS One*. 2013;8(5):e62289.
10. ZhuangY,DengH,SuY,etal.Aptamer-functionalized and backbone redox-responsive hyperbranched polymer for targeted drug delivery in cancer therapy. *Biomacromolecules.* 2016;17(6):2050–2062.
11. Zhang F, Correia A, Mäkilä E, et al. Receptor-mediated surface charge inversion platform based on porous silicon nanoparticles for efficient cancer cell recognition and combination therapy. *ACS Appl Mater Interfaces*. 2017;9(11):10034–10046.
12. Li X, Zhu X, Qiu L. Constructing aptamer anchored nanovesicles for enhanced tumor penetration and cellular uptake of water soluble chemotherapeutics. *Acta Biomater*. 2016;35:269–279.
13. Jain A, Kesharwani P, Garg NK, et al. Galactose engineered solid lipid nanoparticles for targeted delivery of doxorubicin. *Colloids Surf B Biointerfaces*. 2015;134:47–58.
14. Chen C, Hu H, Qiao M, et al. Anti-tumor activity of paclitaxel through dual-targeting lipoprotein-mimicking nanocarrier. *J Drug Target*. 2015;23(4):311–322.
